# Improved Dynamic Postural Task Performance without Improvements in Postural Responses: The Blessing and the Curse of Dopamine Replacement

**DOI:** 10.1155/2012/692150

**Published:** 2011-12-08

**Authors:** K. B. Foreman, C. Wisted, O. Addison, R. L. Marcus, P. C. LaStayo, L. E. Dibble

**Affiliations:** Department of Physical Therapy, College of Health, University of Utah, 520 Wakara Way, Salt Lake City, UT 84108, USA

## Abstract

*Introduction*. Dopamine-replacement medications may improve mobility while not improving responses to postural challenges and could therefore increase fall risk. The purpose of this study was to measure reactive postural responses and gait-related mobility of patients with PD during ON and OFF medication conditions. *Methods*. Reactive postural responses to the Pull Test and performance of the Functional Gait Assessment (FGA) were recorded from 15 persons with PD during ON and OFF medication conditions. *Results*. Persons with PD demonstrated no significant difference in the reactive postural responses between medication conditions but demonstrated significantly better performance on the FGA when ON medications compared to OFF. *Discussion/Conclusion*. Dopamine-replacement medications alone may improve gait-related mobility without improvements in reactive postural responses and therefore could result in iatrogenic increases in fall risk. Rehabilitation providers should be aware of the side effects and limitations of medication treatment and implement interventions to improve postural responses.

## 1. Introduction

Parkinson disease (PD) is the most prominent of the hypokinetic disorders [1, 2]. The cardinal features of PD are tremor at rest, rigidity, hypokinesia, and postural instability [3, 4]. Postural instability and falls constitute major reasons for the serious complications in advanced PD [5, 6]. Falls are associated with high morbidity, mortality [7], and diminished quality of life [8, 9]. Current estimates report that up to 70% of those with PD fall each year, and 13% fall more than once a week [5, 10].

The majority of persons with PD will be treated with dopamine-replacement medications and the benefits of these medications on overall motor function and mobility are well established [11, 12]. However, limitations of dopamine replacement do exist. One of these limitations is the minimal effect of dopamine-replacement medications on postural instability [13–15]. Coupling the benefits of increased gait-related mobility and the limitation that postural instability is dopamine-resistant raises the possibility that fall risk may increase through increased exposure to postural challenges. With such a high incidence of falls and the apparent dopamine-resistant nature of postural instability, an understanding of the extent and character of how postural responses and gait-related mobility respond to dopamine-replacement medication is critical for optimal rehabilitative treatment.

Despite the apparent paradox between dopamine replacment effects on postural responses and gait-related mobility, to our knowledge, no studies have systematically examined these variables in detail. As an intial step in exploring this postural response—mobility paradox, we sought to examine the potential differential effect of dopamine replacement on postural instability and gait-related mobility. This study had the following objectives: (1) quantitatively measure the kinematic characteristics of reactive postural responses and gait-related mobility in persons with PD during both ON and OFF medication conditions and (2) examine the specific components of gait-related mobility (e.g., on level surface, speed, with change in head position, with pivots, over obstacle, with narrow support, with eyes closed, backwards, and steps) that were dopamine-responsive. Based on previous research [13, 14], we hypothesized that dopamine replacement would not improve the kinematics of reactive postural responses. In contrast, we hypothesized that dopamine replacement would improve performance on gait-related mobility, but only through the improvement of specific components of the Functional Gait Assessment (FGA).

## 2. Methods

### 2.1. Selection of Participants

Potential participants were a sample of convenience recruited through referral from local neurologists or response to advertisement in a PD support group newsletter. The inclusion criteria were a medically confirmed diagnosis of idiopathic PD, a stable and neurologist-optimized medication regime that included dopamine replacement as well as other anti-Parkinson medications, and the ability to independently ambulate in the community with or without an assistive device. PD participants were excluded from the study if they had a history of medical conditions (orthopedic, cardiovascular, or otherwise) that would limit their ability to participate in the study procedures.

### 2.2. Measures

The most common research paradigms to examine medication effects on postural instability utilize sliding or rotating force plates that induce postural sway. While having high degrees of internal validity for research purposes, these paradigms lack external and ecologic validity because the floor sliding or rotating underneath a person is not commonly encountered in daily life. Additionally, many of these studies limit their analysis to the components of sway while the base of support remains fixed omitting analysis of protective steps [11, 16]. Therefore, rather than using measures that lacked ecological validity, we selected the Pull Test because of its wide use in clinical neurology practice. Clinically, the Pull Test became the most widely used tool for clinical evaluation of postural instability in patients with PD when it was incorporated into the Unified Parkinson Disease Rating Scale (UPDRS) [17] in 1987. However, current research suggests the Pull Test in isolation is not accurate in predicting fallers, especially in the ON medication state [5, 18]. Also, the Pull Test has no formal consensus on its exact execution and low intra- and interrater consistency [5, 19]. Despite these concerns, the Pull Test is one of the only clinical balance test that examines reactive postural responses and provides insight into postural reflexes without being confounded by other aspects of mobility [7]. In order to examine postural responses, without being corrupted by mobility, the Pull Test is performed by pulling the subject's shoulders posteriorly inducing a protective stepping response. To our knowledge, no studies have kinematically examined the Pull Test to explore the temporal and spatial characteristics in response to interventions such as dopamine replacement.

Ideally, community ambulation and monitoring of fall risk would provide direct measurement of gait-related mobility including step counts [20], variability of ambulatory activity [21], episodes of instability, and falls. Although some research groups have demonstrated monitoring within limited tasks or environments [22, 23], sustained multiday measurement is not technologically feasible at this time and is subject to a multitude of confounding influences [24]. Because of these concerns, we selected a clinical measure that is comprised of a set of posturally challenging gait tasks that a person with PD may encounter during community mobility (the FGA [25]). Previous research has suggested that the FGA may have greater ecological validity to postural challenges during community mobility than the Pull Test [26–28]. Furthermore, the FGA was selected because previous research has documented its validity in people with Parkinson disease [18, 29], vestibular disorders [25], as well as other neurologically impaired populations [30]. The FGA was administered in a standardized location as described in the original publication [25] and is comprised of 10 items each worth a maximum of 3 points for a total possible score of 30. Higher scores are indicative of more stability during-specific balance tasks.

### 2.3. Procedures

Prior to testing, approval for the study was obtained from the Institutional Review Board (IRB) at the University of Utah. After recruitment, the purposes and procedures of the study were explained and all subjects signed an IRB approved consent form. After obtaining consent, demographics and disease specific variables were obtained from each participant.

All testing was conducted at the Wellness and Rehabilitation Clinic and the Motion Capture Core Facility at the University of Utah, Department of Physical Therapy, and took place on two separate days.

For both days of testing, the clinically defined OFF medication condition was induced by having the participant off their dopamine-replacement medications for at least 12 hours prior to testing and is consistent with CAPIT guidelines for OFF medication testing [31]. After completing OFF medication testing, participants took their medication and rested for 1 to 1.5 hours and were retested in a clinically defined ON medication condition.

On the first testing day, the motor subsections of the UPDRS and FGA, during both ON and OFF medication conditions, were conducted by one physical therapist that had undergone standardized training on performance of the UPDRS. Because of the significant medication effects, the tester was not blinded to medication condition. In conjunction with the UPDRS testing, a modified Hoehn and Yahr (H&Y) stage [32] was assigned and a single Pull Test was performed and rated using the standardized scoring criteria [17]. Following completion of the UPDRS, participants performed the FGA.

On the second day, testing was performed in the Motion Capture Core Facility. This laboratory is equipped with an eight-camera motion analysis system (Vicon Motion Systems; Oxford, UK) and two force plates (AMTI; Watertown, Mass, USA). Prior to participants' entry into the laboratory, a static and dynamic calibration of the system was performed. Individual anthropometric data were recorded. Passive reflective markers were placed on bony landmarks utilizing a standardized gait analysis marker set (Plug-In-Gait, Vicon Motion Systems; Oxford, UK). Following subject and system preparation, participants were given an explanation of the Pull Test prior to the execution of the test trials [17]. Once the participant gave verbal confirmation that they understood the test, the participant was placed into position. The examiner, using the UPDRS testing description, performed the Pull Test. Participants performed five trials in both the ON and OFF medication condition. For all trials, kinetic and kinematic data were collected at 250 Hz.

Performance of the Pull Test was characterized using select spatial and temporal variables rather than just using the observational criteria as outlined in the UPDRS. To accomplish this, we segregated out 5 potential temporal and spatial contributors to abnormal Pull Test performance. These variables were chosen to specifically examine temporal and spatial constructs that have been previously shown to be affected by PD (reaction time, movement amplitude, and movement speed) [16]. The five kinematic dependent variables were defined as follows.

- *Step reaction time*: the time latency (in seconds [sec]) from the initial examiner induced shoulder movement until the time of initial foot movement of the initial stepping limb.
- *Step length*: the distance (in centimeters [cm]) from the static sagital plane position of the heel marker of the initial stepping limb to the sagital plane position of the heel marker at initial contact of the initial stepping limb.
- *Step average velocity*: step length divided by step time (in cm/sec). Step time was defined as the time latency from initial foot movement until the time of foot contact (in sec) of the initial stepping limb.
- *COM displacement*: the sagital plane distance (in cm) from the initial COM position to the COM position at time of foot contact of the initial stepping limb.
- *COM average velocity*: COM displacement divided by COM time (in cm/sec). COM time is defined as time latency from initial COM movement until the time of foot contact (in sec) of the initial stepping limb.

For each dependent variable, the average of the first three fully measured trials was used as the representative dependent variable. A fully measured trial consisted of the participant taking at least one step backwards to regain balance following the Pull Test and that all markers remained visible during the trial.

### 2.4. Data Analysis

All statistical analyses were performed with SPSS 16 for Macintosh (SPSS Inc.). Descriptive statistics were performed for demographic variables. The independent variable used for analysis of our primary hypotheses was medication condition (2 levels: ON and OFF medication). Due to the relatively small sample size and the potential for nonnormally distributed data, in the primary analyses, between medication condition differences were compared using separate nonparametric tests for dependent samples.

To examine our findings in more detail, we performed several post hoc means of analysis. First, between-condition effect sizes were calculated to compare the magnitude of effect of the kinematic variables and the FGA. In addition, we examined the changes of the specific items on the FGA in order to gain insight into the locus of effect of medication on FGA performance. Differences between the ON and OFF medication conditions for each FGA item were compared using separate nonparametric tests for dependent samples and between-condition effect sizes. A determination of whether or not an item was dopamine-responsive was made by examining the statistical significance, the within-medication effect size, and the number of individuals in the sample who improved on an item when ON medication. A conservative approach was applied to this decision in that items were determined to be dopamine-responsive only if 3 criteria were met: (1) there was statistical significance between medication conditions (*P* < 0.005), (2) there was a large effect size (ES > 0.70), and (3) the majority of individuals tested demonstrated a performance improvement with dopamine-replacement medication (>7/15).

The experiment wide level of significance was set at *P* < 0.05. However, to control for type I error risk, the overall alpha level for the tests for differences was adjusted using a Bonferroni correction separately within the primary and post hoc analyses (primary analyses: 0.05/6 comparisons, therefore *P* < 0.008 was needed for significance on individual kinematic variables, and the overall FGA; post hoc analyses: 0.05/10, therefore *P* < 0.005 was needed for significance on individual FGA items).

## 3. Results

Fifteen persons (9 male, 6 female; mean age: 67 ± 13 years) with PD (disease duration: 7.5 ± 5.0 years) participated in this study. Their median (range) Hoehn and Yahr rating and mean (SD) UPDRS (motor subsection) was 2.5 (2–4) and 13.7 (6.8), respectively, while ON medication and 3.0 (2.5–4) and 27.6 (7.0), respectively, while OFF medication. Furthermore, 8 of the 15 participants in this study reported a history of falls.

### 3.1. Comparison of Reactive Postural Responses during ON and OFF Medication Conditions

**Figure 1:**
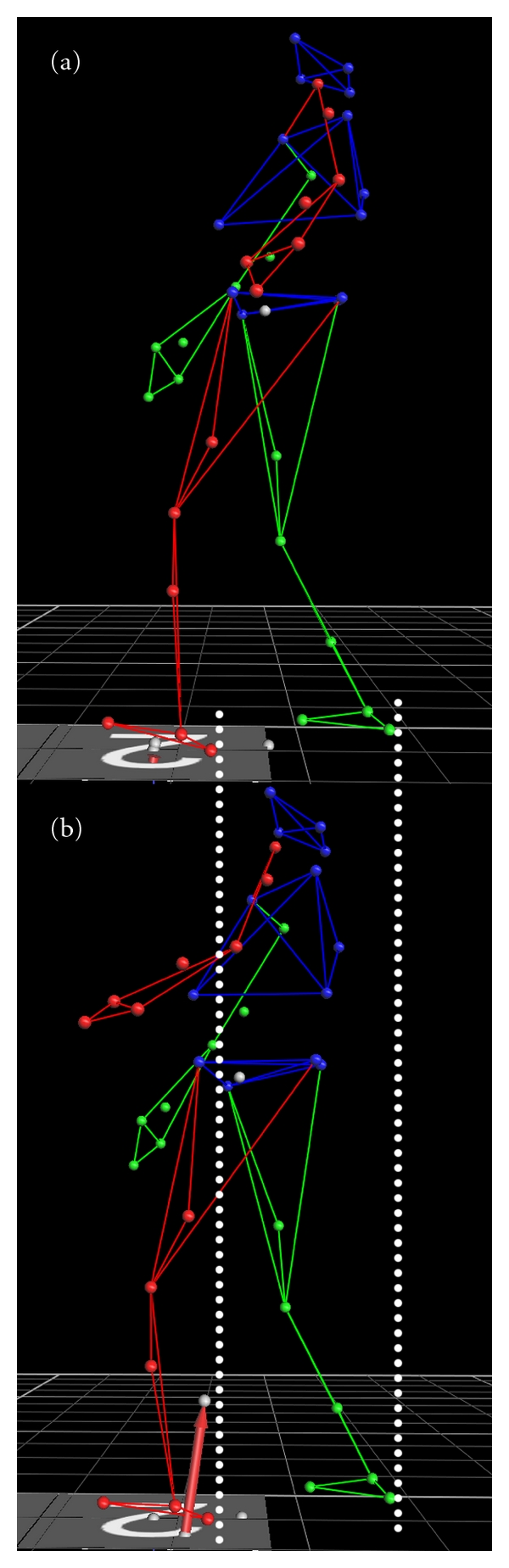
Visual representation of marker data during ON (a) and OFF (b) medication testing trials. White lines depict the equality of step length (Mean (SD): (a) 25.94 (10.33), (b) 25.72 (11.61)).

**Figure 2:**
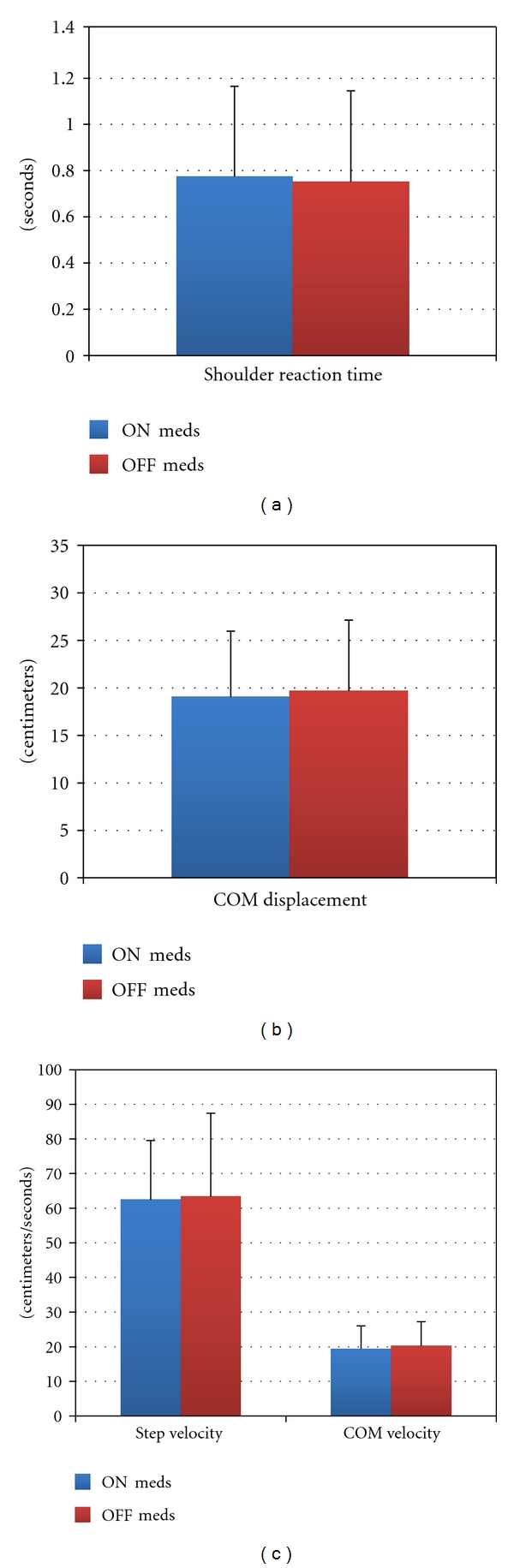
Postural response variables.

Comparison of the reactive postural response variables recorded from the Pull Test revealed no significant difference between ON and OFF medication conditions. In addition, the effect sizes for dopamine replacement for all the postural response variables were small (0.02–0.12) (Table 1, Figures 1 and 2).

### 3.2. Comparison of Clinical Balance Test Performance during ON and OFF Medication Conditions

**Figure 3:**
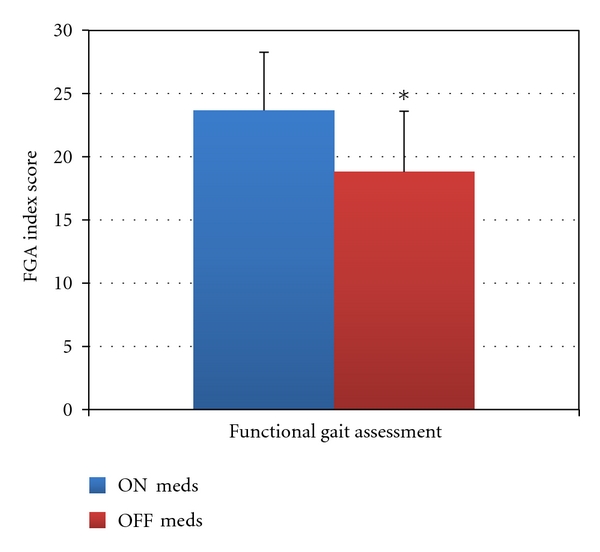
Clinical balance test results. *P ≤ 0.008.

Comparison of the index scores for the FGA revealed a significant higher score during the ON medication condition (*P* ≤ 0.008). Furthermore, the effect size for dopamine replacement on the FGA score was 1.07 (Table 1, Figure 3). In addition, post hoc examination revealed that dopamine-replacement-medication-induced improvements in FGA scores were focused on a select group of tasks (Table 2).

## 4. Discussion

Our clinical experience and previous reports in the literature have suggested that dopamine replacement may have a differential effect on reactive postural responses compared with gait-related mobility. Specifically, through its reduction of bradykinesia and rigidity [33, 34], it may improve gait-related mobility. Despite these improvements, laboratory studies of reactive and anticipatory postural tasks suggest that postural coordination is not improved [13, 35]. Therefore, with improved gait-related mobility and deficient postural coordination, some individuals may have an increased risk of falling. This paradox was the basis for this study.

Our results agreed with our hypotheses that dopamine replacement does not have a significant influence on reactive postural responses as measured by the temporal and spatial characteristics of the Pull Test. In addition, as hypothesized, dopamine-replacement medication improved gait-related mobility as measured by the overall FGA score. Further investigation of the results from the FGA indicated that dopamine-replacement medication improved a limited number of items.

Ultimately, fall events in everyday life are a product of postural abilities and the frequency of exposure to postural challenges. The research designs (ON and OFF medication testing as well as the measures utilized) were intended to systematically provide an initial controlled examination of the possibility that dopamine-replacement medications may improve gait-related mobility without commensurate improvements in reactive postural responses. As an intial step in exploring this postural response—mobility paradox, we found that this is indeed the case. Conceivably, if such a differential effect persisted during community mobility, it could lead to increased fall risk and falls in the community through greater exposure to balance challenges and still deficient postural responses. Certainly, this proposition requires further research.

## 5. A Measured View of the Pull Test

The validity of the Pull Test as a predictor of falls and value in clinical balance examinations has been questioned [18, 36, 37]. Although our results could be seen as support for this view, we do not interpret our findings in this way. The kinematic characteristics of the Pull Test reported in this study are consistent with the hypokinetic reactive postural responses seen in other studies [14, 27]. Few clinical balance tests examine reactive postural responses as a component of the motor sign of postural instability. In isolation, such information provides a narrow view of potential contributors to fall risk of persons with PD in the community. However, in conjunction with other clinical balance tests, the examination of reactive postural responses may provide clinicians with a better understanding of postural instability and fall risk in persons with PD. In addition, concerns regarding Pull Test reliability may be addressed through the use of the recently proposed Push and Release Test [37] as well as the Balance Evaluation Systems Test (BESTtest and a streamlined version (the Mini-BEST)) [29, 38].

## 6. Implications for Rehabilitation

Through the analysis of the validity indices of clinical balance tests, we previously advocated for a battery of tests [39] and environmentally valid testing [18] in the examination of fall risk in individuals with PD. Our current findings add an additional dimension to this issue. Analysis of reactive postural responses revealed no consistent medication effect. Examination of specific FGA items suggested that tasks with stable sensory integration demands (e.g., walking on solid ground with eyes open) were more likely to be dopamine-responsive. In contrast, the dopamine-nonresponsive items shared the constraint of fluctuating sensory integration demands (e.g., gait with horizontal head turns). While this interpretation is speculative, such findings suggest that clinicians should not blindly accept a composite score or specific biomechanical outcome as an indicator of fall risk or as response to a rehabilitation intervention. Rather, there must be a critical analysis of the individual task performance in order to understand the clinical implications of examination findings and the potential targets for intervention.

Despite the fact that postural instability appears to be a dopamine-resistant motor sign, it does not follow that it is not amenable to change. There are few studies that have examined the efficacy of focused rehabilitation interventions on kinematic and kinetic outcomes [40]. In the few studies that have examined such outcomes, there are suggestions that reactive postural responses or postural sway may improve with focused training of an adequate dosage [41].

## 7. Limitations and Directions for Research

Despite their statistical significance, these results should be interpreted with caution. Future research with larger samples is needed to gain further insight into the beneficial and potentially detrimental effects of dopamine replacement on postural performance and falls. Furthermore, this study included only persons currently taking dopamine-replacement medications, and we did not randomize the order of the ON and OFF medication conditions. While such a cohort may reflect persons who have progressed to a moderate disease severity, persons with mild PD (Hoehn and Yahr stage 1) and severe PD (Hoehn and Yahr stage 5) did not participate in this study. Future research should examine participants with these characteristics as well as persons who have undergone surgical management of their PD (such as deep brain stimulation). Lastly, by design, this study used constrained outcomes, such as the Pull Test and the FGA, as an initial test of the posture and mobility paradox. Future studies of postural performance and falls in persons with PD should attempt to employ validated measures of reactive and anticipatory balance responses, clinical balance abilities, and community ambulatory/fall risk monitoring as outcomes.

## 8. Summary and Clinical Implications

Our findings suggest that dopamine-replacement medications alone may improve gait-related mobility without commensurate improvements in reactive postural responses and therefore could result in iatrogenic increases in fall risk. Rehabilitation providers should be aware of the limitations of dopamine-replacement treatment and implement interventions intended to improve postural responses.

## Figures and Tables

**Table 1:** Results of PD group ON and OFF medication (Mean ± SD).

	Step reaction time (sec)	Step length (cm)	Step avg velocity (cm/sec)	COM displacement (cm)	COM avg velocity (cm/sec)	Pull Test (UPDRS motor subsection item 30)	FGA
ON meds 95% CI	0.77 ± 0.39	25.94 ± 10.33	62.45 ± 17.11	19.05 ± 6.91	19.42 ± 6.59	0.73 ± 0.46	23.67 ± 4.59*
	0.56–0.99	20.22–31.67	52.98–71.93	15.23–22.88	15.77–23.07	0.48–0.99	21.12–26.21
OFF meds 95% CI	0.75 ± 0.39	25.72 ± 11.61	63.38 ± 24.05	19.66 ± 7.46	20.20 ± 7.01	1.0 ± 0.53	18.80 ± 4.80
	0.53–0.97	19.29–32.15	50.07–76.70	15.53–23.80	16.32–24.09	0.70–1.30	16.14–21.46
Effect size	0.06	0.02	.05	0.09	0.12	0.56	1.07

*P ≤ 0.008.

**Table 2:** FGA item analysis: items were determined to be dopamine responsive if 3 criteria were met: (1) there was statistical significance between medication conditions, (*P* < 0.005) (2) there was a large effect size (ES > 0.70), and (3) the majority of individuals tested demonstrated a performance improvement with dopamine replacement.

FGA Item	Between-medication condition significance level	Between-medication condition effect size	Number with positive dopamine-replacement effect	Dopamine-responsive (Yes/No)
(1) Gait on a level surface	*P* < 0.002	1.07	9/15	Yes
(2) Change in gait speed	*P* < 0.004	1.03	8/15	Yes
(3) Gait with horizontal head turns	*P* < 0.017	0.63	5/15	No
(4) Gait with sustained vertical head positions	*P* < 0.003	0.85	7/15	No
(5) Gait and pivot turn	*P* < 0.017	0.65	7/15	No
(6) Step over obstacle	*P* < 0.090	0.31	2/15	No
(7) Gait with narrow base of support	*P* < 0.003	0.90	9/15	Yes
(8) Gait with eyes closed	*P* < 0.048	0.47	5/15	No
(9) Ambulating backwards	*P* < 0.017	0.75	7/15	No
(10) Steps	*P* < 0.080	0.32	2/15	No
